# Supplementary material for: Identifying expanding TCR clonotypes with a longitudinal Bayesian mixture model and their associations with cancer patient prognosis, metastasis-directed therapy, and VJ gene enrichment
Source: Bioinformatics. 2026 Sep 2;42(9):btag659. doi: 10.1093/bioinformatics/btag659 (PMC13614417; doi:10.1093/bioinformatics/btag659)
Supplement: Supplementary file 1 [file btag659_supplementary_data.pdf]

Supplementary Material: Identifying expanding TCR clonotypes with a longitudinal Bayesian mixture model and their associations with cancer patient prognosis, metastasis-directed therapy, and VJ gene enrichment

David Swanson, Alexander Sherry, Cara Haymaker, Alexandre Reuben, Chad Tang

A1 Simulation

We performed several simulations to examine whether the fitting procedure recovers the parameters under which samples were generated. We considered the 100 samples of logged values of α_i and β_i as distributed Gaussian with mean -0.5 or -0.3 and 6.0 or 4.4, respectively. The standard deviations under these parameterizations were 0.5 or 0.3, respectively. Under subsequent exponentiation, these parameters yield approximately the same mean for the Gamma prior, but with the pairing of -0.3 and 4.4 having an on average higher variance from which the Poisson mean parameter is sampled. Intuitively this should yield more information longitudinally with respect to the clone's dynamic or static component membership. We generated 60,000 total clones on 2 or 3 followup times depending on the simulation, 20 percent of which were generated under the dynamic model and 80 percent under the static. For the 3 followup simulation, approximately 34% of samples had some missingness at the 2nd or 3rd followup time. Offset terms were generated under a Gamma distribution with shape parameter of 5000/20 and rate parameter 1/20.

The model was fit with combinations of 2 or 3 followups, in the latter case with missingness, and the α_j and β_j parameterizations of the Gamma distribution with smaller or bigger variance. We used Hamiltonian Monte Carlo (HMC) via STAN for model fitting, with 2500 samples on 2 independent chains with a burn-in of 500 [31]. An advantage of Hamiltonian Monte Carlo is samples are nearly independent so one more quickly achieves a sampling error that is an order of magnitude smaller than estimation error.

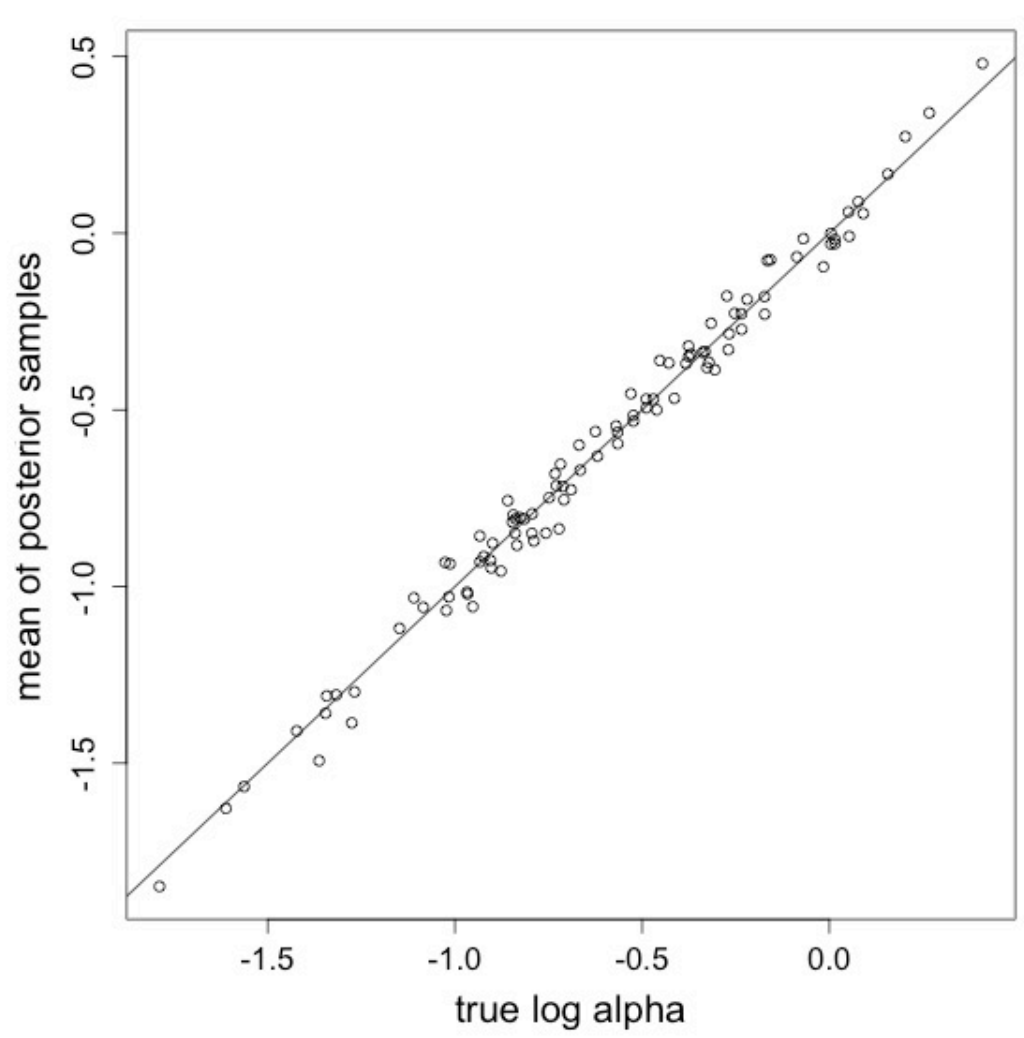

(a) Small variance simulation for α on 3 followups. The standard deviation for samples of each $\log \alpha_j$ was approximately 0.055 and therefore a 95% interval around each corresponds to slightly less than half the tick mark length.

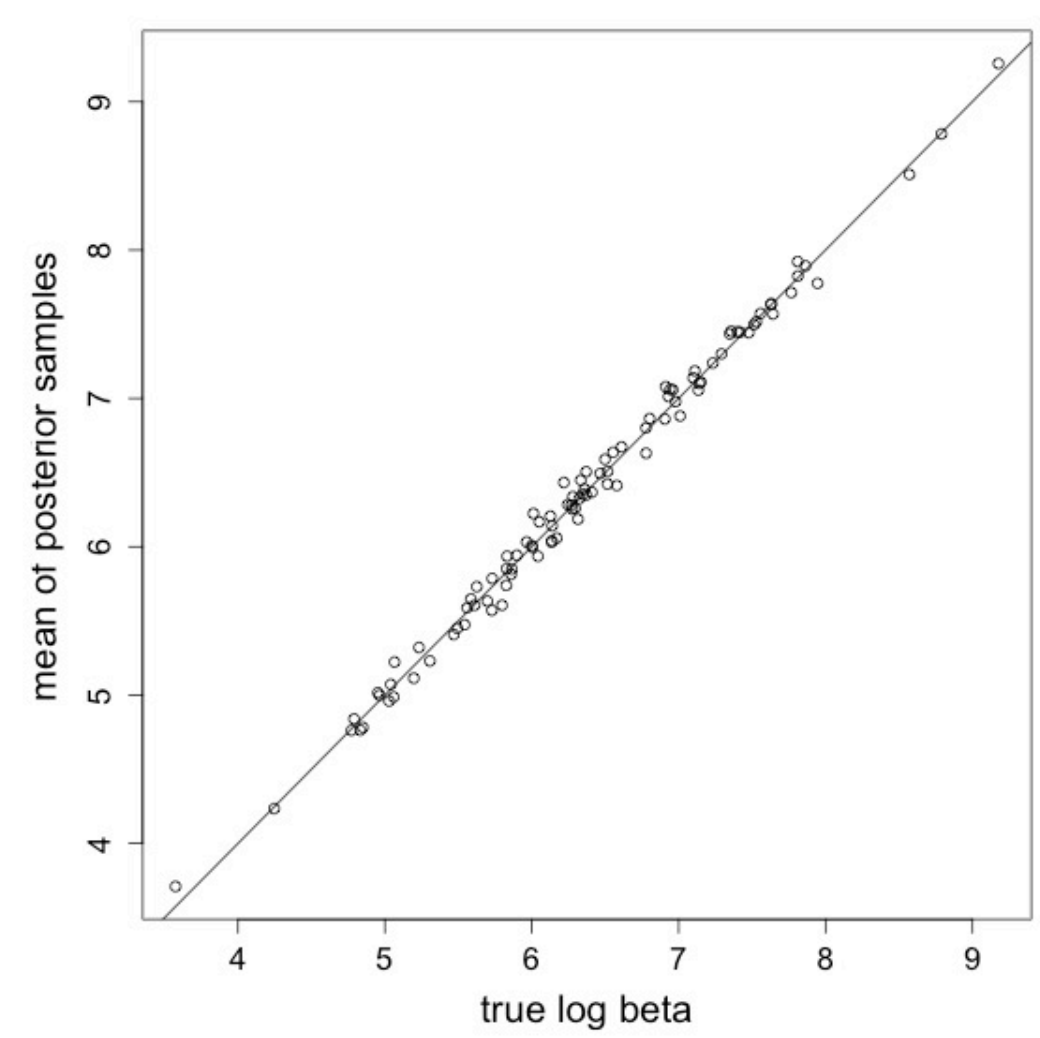

(b) Small variance simulation for β on 3 followups. The standard deviation for samples of each $\log \beta_j$ was approximately 0.07 and therefore a 95% interval around each corresponds to slightly less than half the tick mark length.

Figure A1: True parameters versus means of posterior samples, 3 followups. 45 degree line indicates perfect agreement.

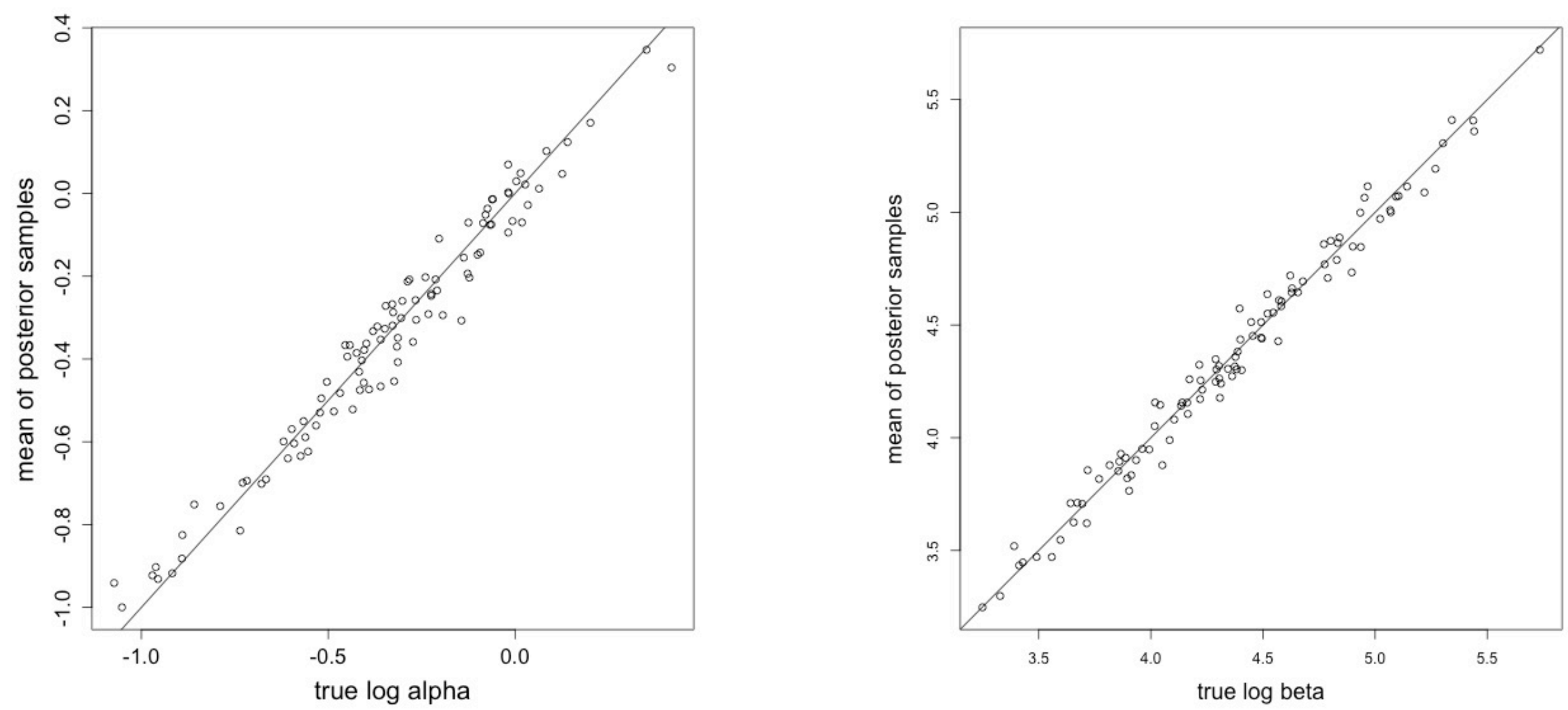

(a) Big variance simulation for α on 2 followups. The standard deviation for samples of each $\log \alpha_j$ was approximately 0.048 and therefore a 95% interval around each corresponds to slightly less than half the tick mark length.

(b) Big variance simulation for β on 2 followups. The standard deviation for samples of each $\log \beta_j$ was approximately 0.065 and therefore a 95% interval around each corresponds to slightly less than half the tick mark length.

Figure A2: True parameters versus means of posterior samples, 2 followups. 45 degree line indicates perfect agreement.

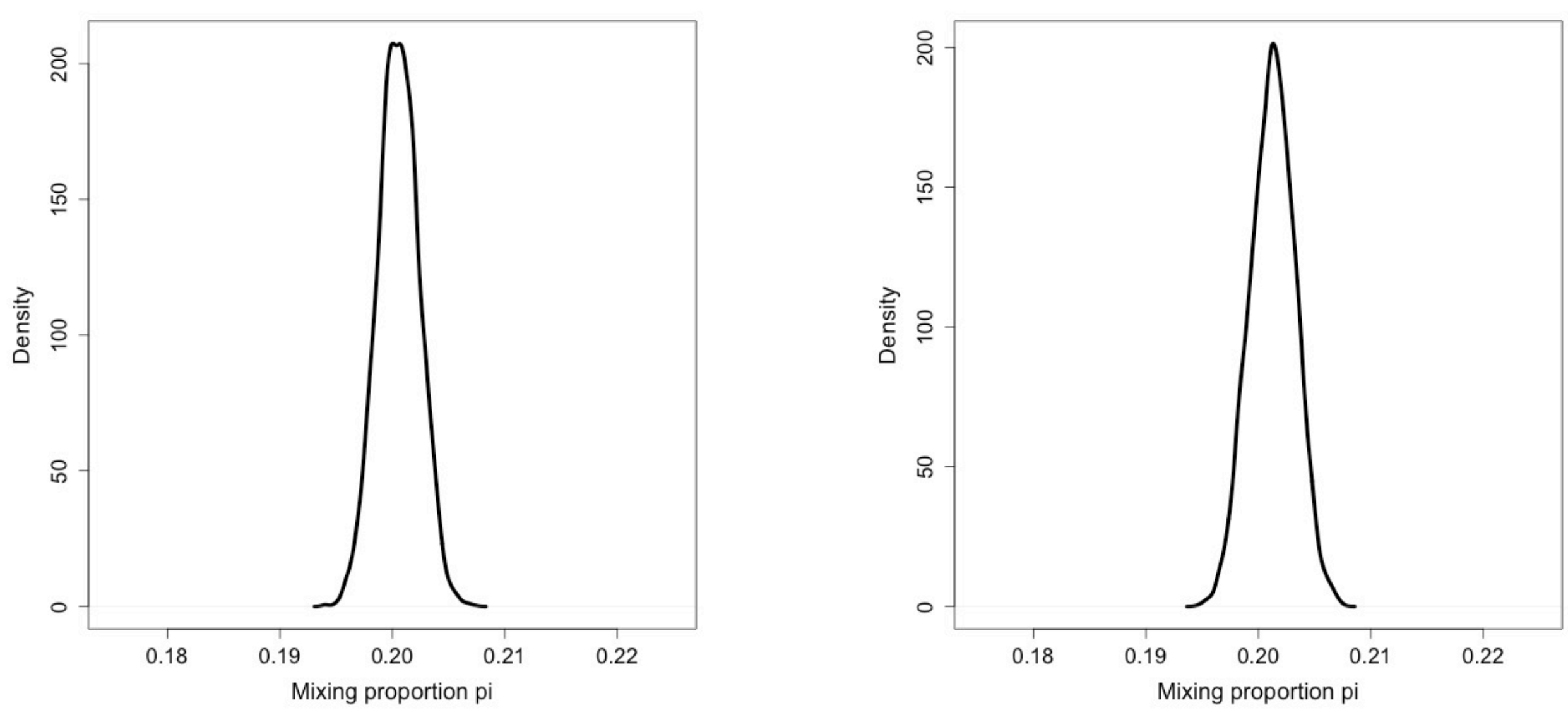

(a) Distribution of samples of π for simulation with larger variance parameterization and on 2 followups.

(b) Distribution of samples of π for simulation with smaller variance parameterization and on 3 followups.

Figure A3: Posterior π samples under different simulation scenarios.

Simulations indicate the fitting procedure characterizes the parameters used in the data generating

mechanism well, with Figures A1 and A2 suggesting tight alignment between true α_i and β_i and the mean of posterior samples. Likewise, Figure A3 suggests samples of logit π align closely under inverse logit transformation to the true parameter of 0.2, with the distribution nearly perfectly centered at the value. The small degree of bias is likely induced by fitting the parameter in symmetric logit space prior to application of the inverse function.

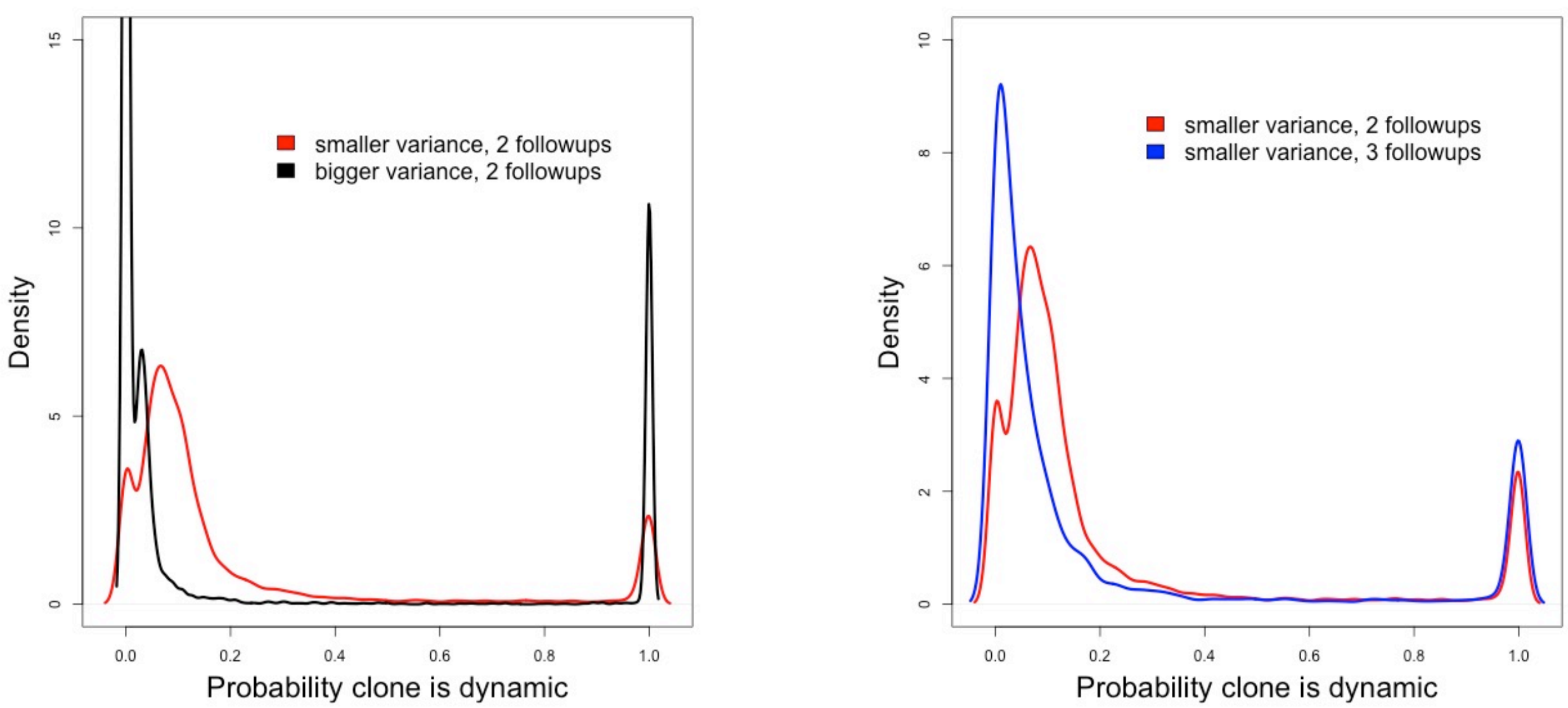

(a) Component membership of clones under 2 followups, bigger and smaller variance.

(b) Component membership of clones under smaller variance, 2 and 3 followups.

Figure A4: Density plots of mean probability component membership under different simulation scenarios.

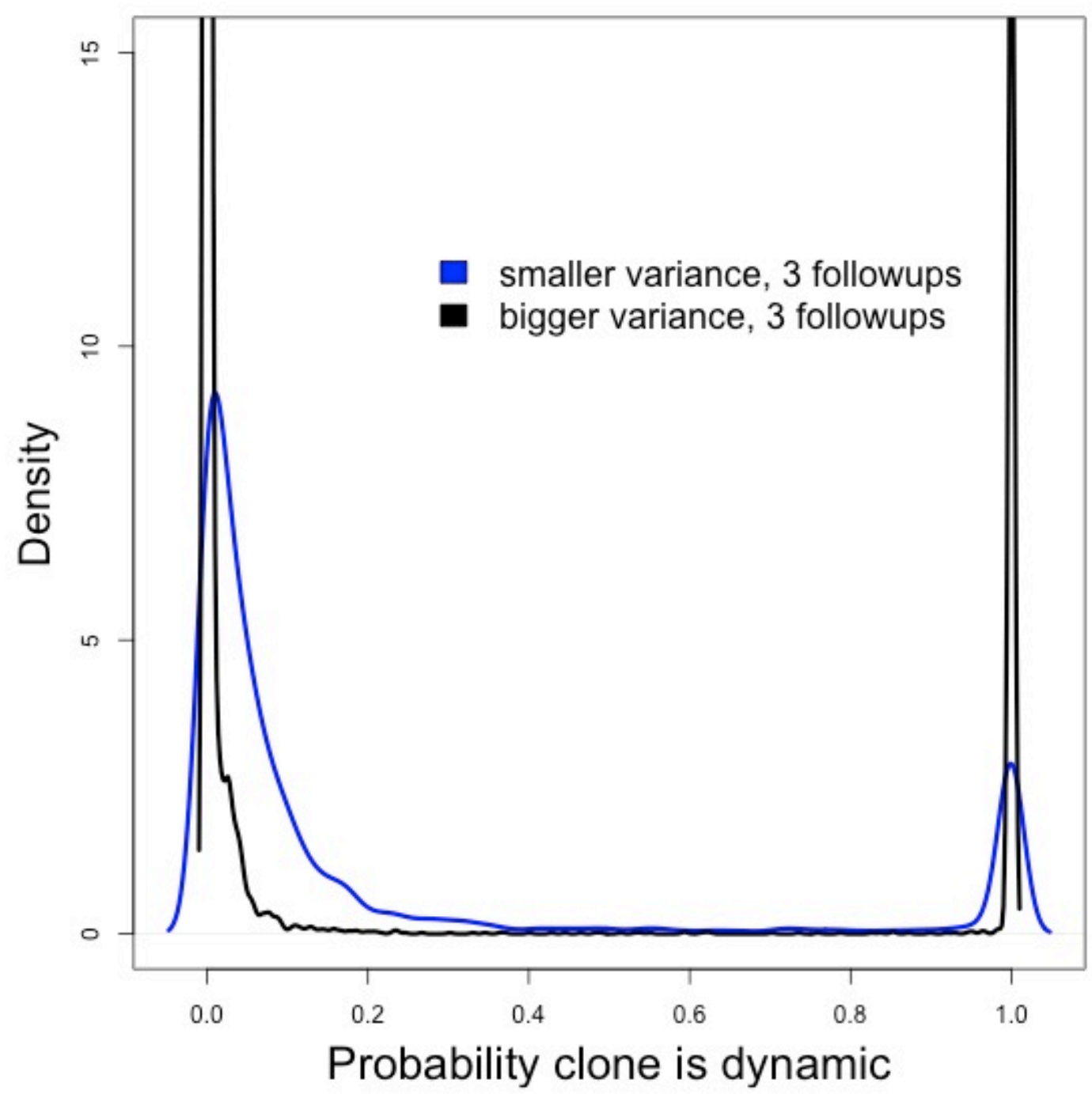

Figure A5: Densities of component membership of clones under 3 followups, smaller and bigger variance.

Figures A4 and A5 likewise show the model discriminating well between static and dynamic clones as intended. The left hand mode in each density plot corresponds to static clones, while the right hand mode corresponds to dynamic. The peakedness of the modes varies by plot as expected–where there is more information to discriminate dynamic versus static behavior with 3 followups rather than 2, the peakedness is greater. Likewise, in the simulation scenarios using a Gamma prior with on average greater variation for the subsequent Poisson mean parameter, the peakedness is greater because dynamic behavior is easier to identify, other things equal.

We additionally explored extreme imbalance in component membership, $\pi \in \{0.001, 0.999\}$, with no influence on the precision with which that parameter nor α, β were estimated. We also misspecified α, β within subject, with implied mean and variance from those parameters at least a factor of 2 greater and less for each, and combinations thereof, with relatively little impact on clone component membership.

A2 Derivation of static and dynamic mixture components

A2.1 Deriving the static model

Since for the static component we have

$$C_{ijk} \,|\, (\lambda_{ij}, O_{jk}, D_{ij} = 0) \sim \text{Pois}(\lambda_{ij} O_{jk})$$

and

$$\lambda_{ij} | (D_{ij} = 0) \sim \text{Gamma}(\alpha_j, \beta_j),$$

then the probability mass function (pmf) for the likelihood and density for the prior are

$$l(C_{ij\cdot} | \lambda_{ij}, O_{j\cdot}) = \prod_{k=1}^{T_j} \frac{\exp(-\lambda_{ij} O_{jk})\,(\lambda_{ij} O_{jk})^{C_{ijk}}}{C_{ijk}!} = \frac{\exp(-\lambda_{ij} \sum_k O_{jk}) \lambda_{ij}^{\sum_k C_{ijk}} \prod_{k=1}^{T_j} O_{jk}^{C_{ijk}}}{\prod_{k=1}^{T_j} C_{ijk}!}$$

$$p(\lambda_{ij}|D_{ij}=0,\alpha_j,\beta_j) = \frac{\beta_j^{\alpha_j}}{\Gamma(\alpha_j)} \lambda_{ij}^{\alpha_j-1} \exp\left(-\beta_j \lambda_{ij}\right),$$

respectively. Taking the product of these terms and integrating out λ_{ij} yields

$$\begin{aligned}
p(C_{ij\cdot}|O_{j\cdot},D_{ij}=0,\alpha_j,\beta_j) &= \int p(C_{ij\cdot},\lambda_{ij}|O_{j\cdot},D_{ij}=0,\alpha_j,\beta_j)\,d\lambda_{ij} = \int l(C_{ij\cdot}|\lambda_{ij},\,O_{j\cdot})p(\lambda_{ij}|D_{ij},\alpha_j,\beta_j) \\
&= \frac{\beta_j^{\alpha_j}\prod_{k=1}^{T_j} O_{jk}^{C_{ijk}}}{\Gamma(\alpha_j)\prod_{k=1}^{T_j} C_{ijk}!} \int_0^\infty \lambda_{ij}^{\sum_k C_{ijk}+\alpha_j-1} \exp\left(-\lambda_{ij}(\beta_j + \sum_k O_{jk})\right) d\lambda_{ij} \\
&= \frac{\beta_j^{\alpha_j}\prod_{k=1}^{T_j} O_{jk}^{C_{ijk}}}{\Gamma(\alpha_j)\prod_{k=1}^{T_j} C_{ijk}!} \cdot \frac{\Gamma(\sum_k C_{ijk}+\alpha_j)}{(\beta_j+\sum_k O_{jk})^{\sum_k C_{ijk}+\alpha_j}} \\
&= \frac{\Gamma(\sum_k C_{ijk}+\alpha_j)}{\Gamma(\alpha_j)\prod_{k=1}^{T_j} C_{ijk}!} \left(\frac{\beta_j}{\beta_j+\sum_k O_{jk}}\right)^{\alpha_j} \prod_{k=1}^{T_j} \left(\frac{O_{jk}}{\beta_j+\sum_k O_{jk}}\right)^{C_{ijk}}
\end{aligned} \tag{2}$$

where we have used conjugacy to evaluate the integral, recognizing the kernel of the Gamma on updated parameters $\alpha_j' = \sum_k C_{ijk} + \alpha_j$ and $\beta_j' = \sum_k O_{jk} + \beta_j$. The integral with respect to λ_{ij} yields the inverse normalizing constant of the Gamma(α_j', β_j').

A2.2 Deriving the dynamic model

For the dynamic component clonal likelihood we have

$$C_{ijk} \mid (\lambda_{ijk}, O_{jk}, D_{ij}=1) \sim \text{Pois}(\lambda_{ijk}O_{jk})$$

and for the prior

$$\lambda_{ijk}|(D_{ij}=1) \sim \text{Gamma}(\alpha_j,\beta_j)$$

so that for each k the pmf and density expressions are

$$l(C_{ijk}|\lambda_{ijk},\,O_{j\cdot}) = \frac{\exp(-\lambda_{ijk}\,O_{jk})\lambda_{ijk}^{C_{ijk}}O_{jk}^{C_{ijk}}}{C_{ijk}!}$$

$$p(\lambda_{ijk}|D_{ij}=1,\alpha_j,\beta_j) = \frac{\beta_j^{\alpha_j}}{\Gamma(\alpha_j)} \lambda_{ijk}^{\alpha_j-1} \exp\left(-\beta_j \lambda_{ijk}\right),$$

respectively. The product across k then is

$$\begin{aligned}
p(C_{ij\cdot} \mid O_{j\cdot},D_{ij}=1,\alpha_j,\beta_j) &= \prod_{k=1}^{T_j} \int l(C_{ijk}|\lambda_{ijk},\,O_{j\cdot})p(\lambda_{ijk}|D_{ij}=1,\alpha_j,\beta_j)\,d\lambda_{ijk} \\
&= \prod_{k=1}^{T_j} \int \frac{\exp(-\lambda_{ijk}\,O_{jk})\lambda_{ijk}^{C_{ijk}}O_{jk}^{C_{ijk}}}{C_{ijk}!} \cdot \frac{\beta_j^{\alpha_j}}{\Gamma(\alpha_j)} \lambda_{ijk}^{\alpha_j-1} \exp\left(-\beta_j\lambda_{ijk}\right) d\lambda_{ijk} \\
&= \int \frac{\exp(-\sum_k \lambda_{ijk}(O_{jk}+\beta_j)) \prod_{k=1}^{T_j} O_{jk}^{C_{ijk}}\,\lambda_{ijk}^{C_{ijk}+\alpha_j-1}}{\prod_{k=1}^{T_j} C_{ijk}!} \cdot \frac{\beta_j^{\alpha_j}}{\Gamma(\alpha_j)}\,d\lambda_{ijk} \\
&= \prod_{k=1}^{T_j} \frac{\Gamma(C_{ijk}+\alpha_j)}{\Gamma(\alpha_j)C_{ijk}!} \left(\frac{\beta_j}{\beta_j+O_{jk}}\right)^{\alpha_j} \left(\frac{O_{jk}}{\beta_j+O_{jk}}\right)^{C_{ijk}}
\end{aligned} \tag{3}$$

where again we have used conjugacy to evaluate the integral, recognizing the kernel of the Gamma on updated parameters $\alpha_j' = \sum_k C_{ijk} + \alpha_j$ and $\beta_j' = \sum_k O_{jk} + \beta_j$. The integral with respect to λ_{ijk} yields the inverse normalizing constant of the Gamma(α_j', β_j').

A3 HLA typing and clonal expansions

HLA typing and baseline-followup data were available for analysis on 23 patients. Results showed modest associations between presence of the A*03 and C*07 alleles and clonal expansions, in the former case positive and the latter negative. Variable HLA cross reactivity and Vβ gene and antigenic preference according to HLA type may in part explain the observed associations [5, 25].

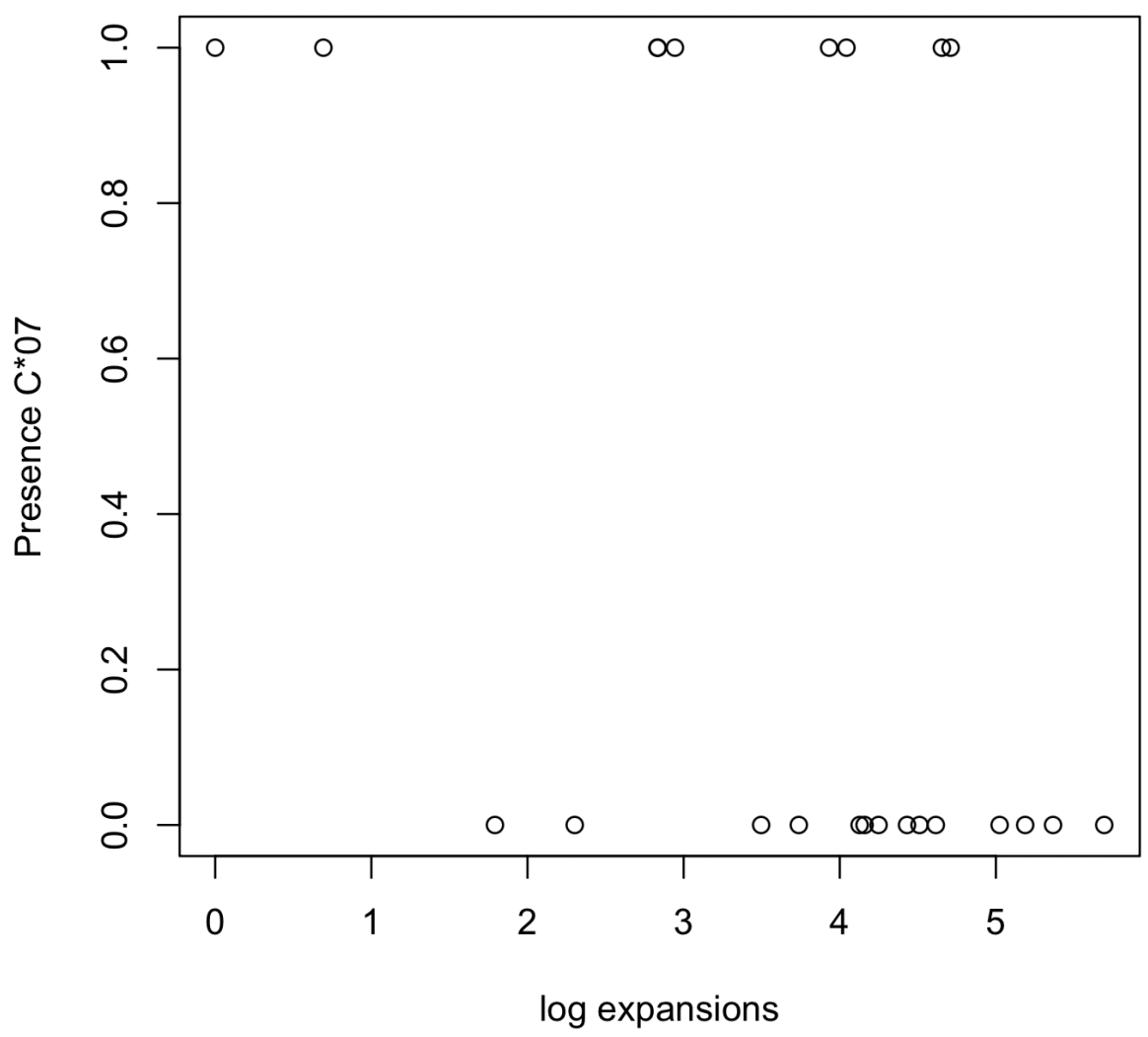

(a) P-value of linear model under log transform, 0.043

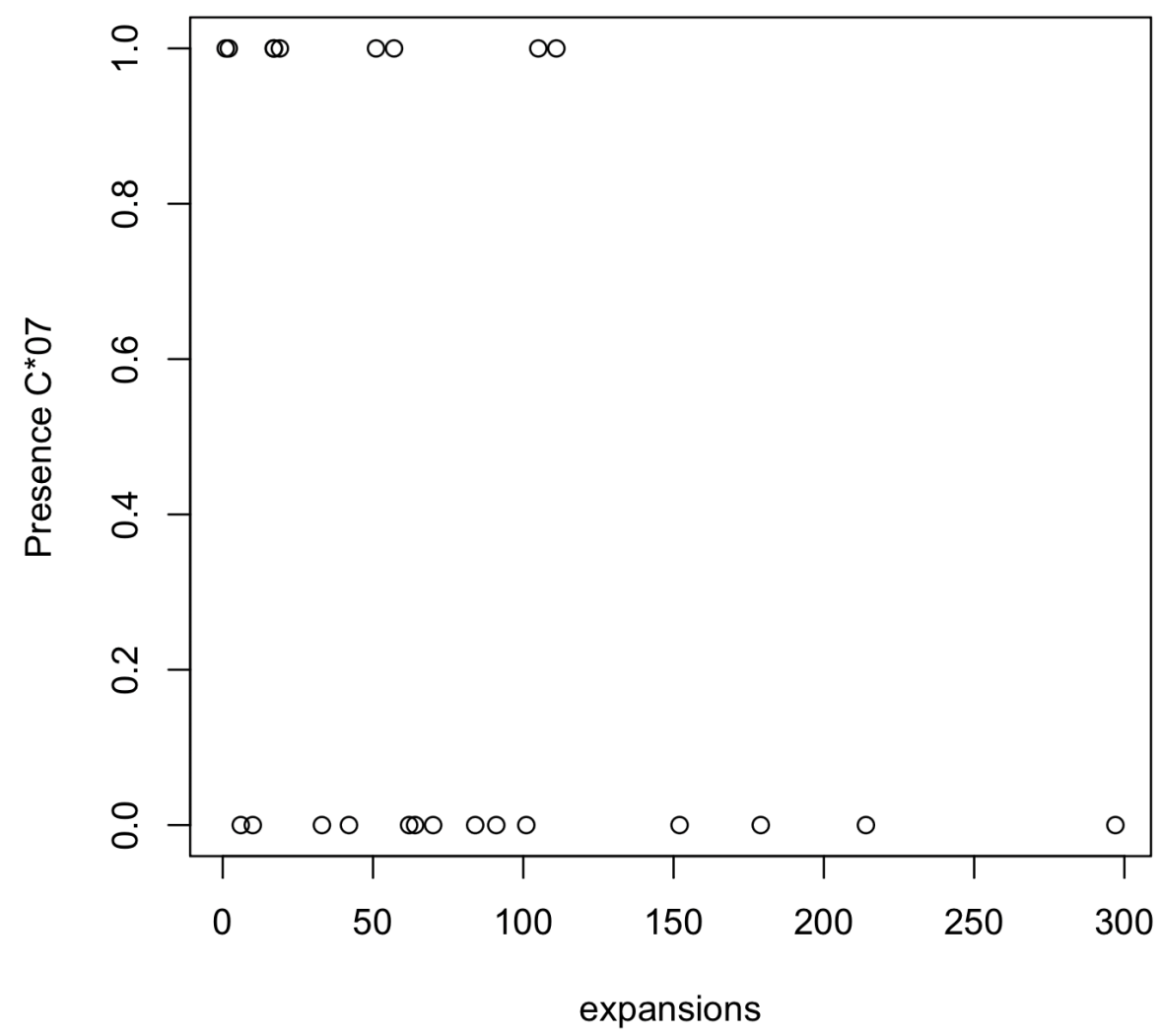

(b) Visual depiction of untransformed association.

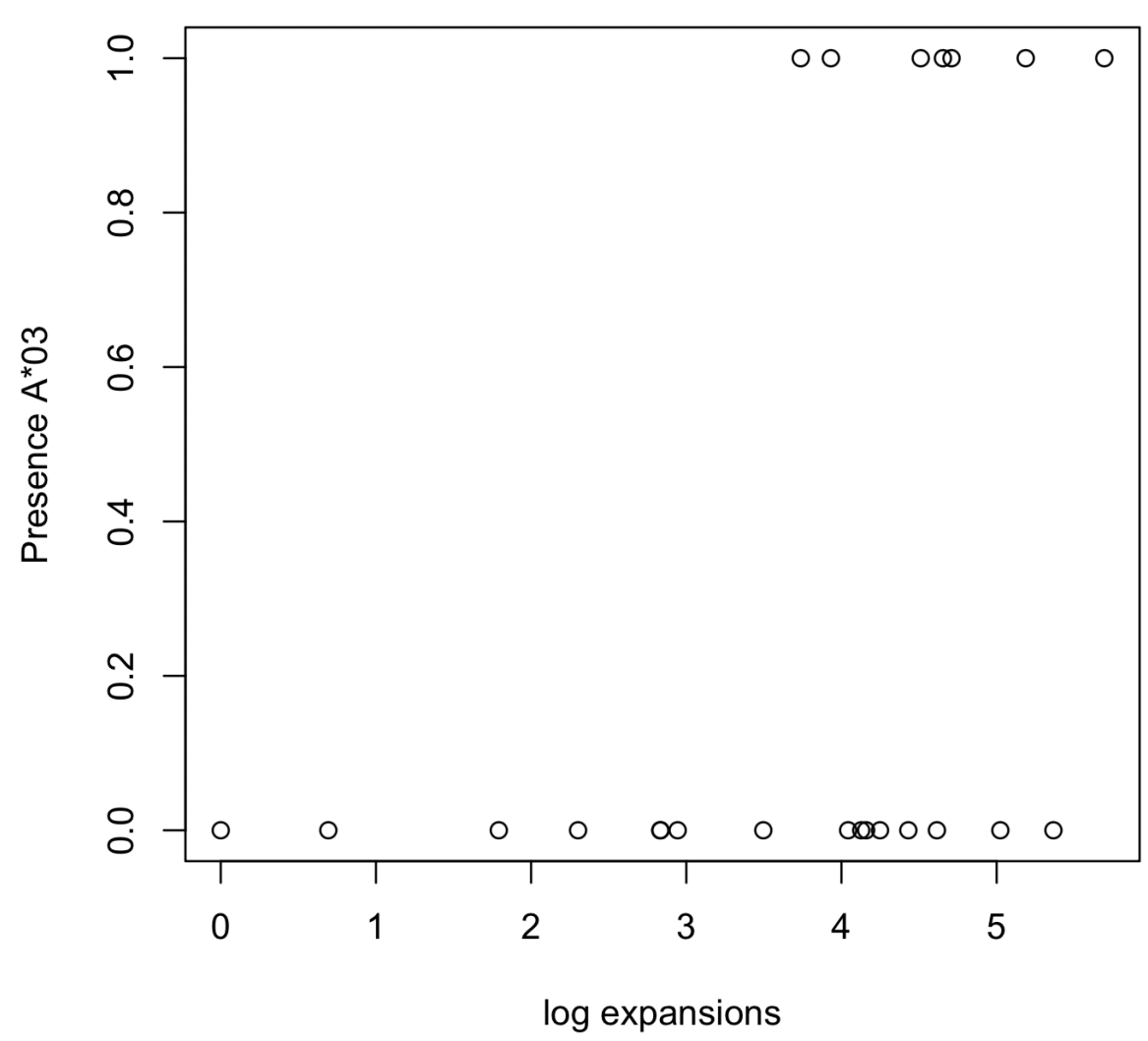

(c) P-value of linear model under log transform, 0.040

Figure A6: HLA type and expansions

A4 Stability selection for VJ gene enrichment and interaction with expansions

Results of stability selection for the VJ gene enrichment analysis and interactions with being an expanding clone. While there is broad overlap of the gene family interactions as compared to the L1 penalized regression model, interactions between gene family and being an expansion are not consistent and may indicate a lack of stability.

	$\hat{\beta}$	95% CI	Z-statistic	Pr(>|Z|)
**Expansion Main Effect**				
Expansion	-2.95	(-3, -2.9)	-144	0
**Gene family interactions**				
TCRBJ02 * TCRBV01	1	(0.34, 1.7)	2.95	0.00314
TCRBJ02 * TCRBV02	-0.14	(-0.22, -0.062)	-3.49	0.000476
TCRBJ02 * TCRBV04	0.0946	(0.022, 0.17)	2.54	0.0111
TCRBJ02 * TCRBV05	0.094	(0.037, 0.15)	3.24	0.00118
TCRBJ02 * TCRBV07	0.155	(0.1, 0.21)	5.73	9.9e-09
TCRBJ02 * TCRBV08	0.0123	(-2.8, 2.8)	0.00868	0.993
TCRBJ02 * TCRBV09	0.219	(0.13, 0.31)	4.82	1.43e-06
TCRBJ02 * TCRBV11	0.439	(0.35, 0.53)	9.56	1.23e-21
TCRBJ02 * TCRBV12	-0.202	(-0.28, -0.12)	-5	5.74e-07
TCRBJ02 * TCRBV17	14.8	(-1900, 1900)	0.015	0.988
TCRBJ02 * TCRBV18	-0.193	(-0.3, -0.082)	-3.43	0.000612
TCRBJ02 * TCRBV19	-0.336	(-0.41, -0.26)	-8.8	1.36e-18
TCRBJ02 * TCRBV24	0.571	(0.42, 0.72)	7.41	1.24e-13
TCRBJ02 * TCRBV26	-14.7	(-1900, 1900)	-0.0152	0.988
TCRBJ02 * TCRBV27	0.134	(0.046, 0.22)	2.99	0.00278
TCRBJ02 * TCRBV28	-0.163	(-0.25, -0.077)	-3.69	0.000222
**Gene family expansion interaction**				
TCRBJ02 * Expansion	-0.0562	(-0.13, 0.02)	-1.45	0.146
TCRBV01 * Expansion	1.48	(0.71, 2.2)	3.78	0.000157

Table A1: Cutoff of 0.45 is used here: VJ gene with expansion interaction identified by log-linear model penalized regression. Intercept and gene family main effects not shown.
